# Real‐World Pharmacological Management of Alcohol Dependence in a Nepalese Rehabilitation Center: Prospective Cohort Study

**DOI:** 10.1002/puh2.70199

**Published:** 2026-03-13

**Authors:** Sanjay Singh, Nirmal Raj Marasine, Sabina Sankhi, Kabita Bhandari, Gajendra Bahadur Bhuju

**Affiliations:** ^1^ Department of Pharmacy CiST College Pokhara University Kathmandu Nepal; ^2^ Department of Pharmacy Shree College of Technology Bharatpur Nepal

**Keywords:** alcohol dependence, Alcohol Use Disorders Identification Test (AUDIT), alcohol withdrawal, Nepal, pharmacological management, Prediction of Alcohol Withdrawal Severity Scale (PAWSS), rehabilitation

## Abstract

**Background:**

Alcohol dependence syndrome (ADS) poses a significant public health issue in Nepal, necessitating effective pharmacological management. This study evaluated the real‐world pharmacological management and short‐term treatment outcomes of ADS patients in a rehabilitation center in Kathmandu, Nepal.

**Methods:**

A prospective cohort study was conducted among 96 adult ADS patients between November 2023 and April 2024. Alcohol consumption severity and withdrawal risk were assessed using validated tools (Alcohol Use Disorders Identification Test [AUDIT] and Prediction of Alcohol Withdrawal Severity Scale [PAWSS]). Treatment regimens, symptom profiles, and liver enzyme levels were documented. Statistical analysis included paired and independent *t*‐tests to assess changes in withdrawal severity and clinical markers, with significance set at *p* < 0.05.

**Results:**

The cohort had a mean age of 37.8 years; 69% were male. At baseline, 96% were identified as problematic drinkers, and all were classified as high risk for withdrawal. Lorazepam (98.8%) and thiamine (97.9%) were the most commonly prescribed agents. A significant reduction in PAWSS scores was observed from admission to follow‐up (7.06 ± 0.89 vs. 3.64 ± 0.94, *p* < 0.001). Liver enzyme levels also improved significantly after treatment. Antipsychotics and benzodiazepines were associated with greater symptom control, whereas antidepressants showed limited impact on withdrawal severity.

**Conclusion:**

Structured, evidence‐informed pharmacological regimens were associated with improved clinical outcomes among ADS patients in this resource‐limited setting. These findings support the integration of standardized ADS protocols in Nepalese rehabilitation centers. Multicenter studies with long‐term follow‐up are warranted.

## Graphical abstract

This prospective cohort study demonstrates that structured, evidence‐informed pharmacological management significantly reduced alcohol withdrawal severity and improved liver enzyme profiles among alcohol dependence patients in a Nepalese rehabilitation center, supporting the implementation of standardized treatment protocols in resource‐limited settings.

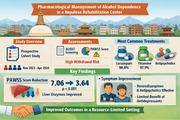

**Abbreviations**
ADS: alcohol dependence syndrome
ALD: alcoholic liver disease
ALP: alkaline phosphatase
AUD: alcohol use disorder
AUDIT: Alcohol Use Disorder Identification Test
AWS: alcohol withdrawal syndrome
BZD: benzodiazepine
DALYs: disability‐adjusted life years
DT: delirium tremens
PAWSS: Prediction of Alcohol Withdrawal Severity Scale
UDCA: ursodeoxycholic acid

## 1 Introduction

Alcohol dependence syndrome (ADS) is a chronic, relapsing disorder marked by a combination of behavioral, cognitive, and physiological changes resulting from repeated alcohol consumption. It often leads to impaired control over drinking, increased tolerance, and the development of withdrawal symptoms [1, 2]. Globally, alcohol use is a significant public health concern, contributing to approximately 3 million deaths annually and accounting for 5.3% of all global deaths. It is also a significant risk factor for various morbidities and premature mortality [3]. In low‐ and middle‐income countries like Nepal, traditional alcoholic beverages such as raksi, jand, and chang are widely consumed, often produced domestically, and closely tied to traditional customs. The social acceptability and affordability of alcohol, along with limited public awareness and a lack of specialized addiction services, contribute to the underdiagnosis and undertreatment of ADS [4, 5]. Moreover, the provisions of the Liquor Control Acts allow informal production and distribution of alcohol, thereby promoting its availability and use.

Clinically, individuals with ADS often present with psychological and physical symptoms, including anxiety, irritability, seizures, tremors, and autonomic instability [6]. Early detection and timely pharmacological intervention are critical in preventing the potentially life‐threatening complications associated with alcohol withdrawal [7]. Tools like the Alcohol Use Disorders Identification Test (AUDIT) and the Prediction of Alcohol Withdrawal Severity Scale (PAWSS) are effective in assessing alcohol use patterns and determining withdrawal severity, thereby aiding in risk assessment and guiding individualized treatment strategies [8, 9]. Pharmacological management plays a pivotal role in treating ADS. Although benzodiazepines (BZDs), including diazepam and lorazepam, are used to control withdrawal symptoms, drugs like disulfiram, naltrexone, and acamprosate are employed for their efficacy in reducing cravings and supporting long‐term abstinence [6, 10]. However, in low‐resource settings like Nepal, the use of these medications remains underexplored due to factors such as cost, availability, and lack of provider training. Despite their proven effectiveness, there is limited documentation on their prescribing patterns and real‐world outcomes in clinical or rehabilitation settings.

Although several studies in Nepal have investigated alcohol use behaviors and the sociocultural context of drinking, few have focused on the pharmacological management of ADS or its outcomes in clinical settings. This lack of evidence hinders the implementation of standardized treatment guidelines that are contextually appropriate. Therefore, this study aimed to evaluate real‐world pharmacological management practices and treatment outcomes for ADS patients at a rehabilitation center in Kathmandu, Nepal.

## 2 Materials and Methods

### 2.1 Study Design, Setting, and Population

A prospective cohort study was conducted from November 2023 to April 2024 at the Amrita Foundation rehabilitation center, Dachi Kathmandu [11]. The site primarily caters to patients with ADS and is well‐equipped with structured facilities and efficient patient management systems. A consultant psychiatrist conducts daily visits to ensure regular check‐ups and follow‐ups for ADS patients as well as others receiving treatment at the center. The facility serves a diverse population in terms of location, age, and gender. Patients are typically admitted for a minimum duration of 3 months, which allows for comprehensive observation and evaluation of their condition while minimizing the risk of subject drop‐out from the study. Patients diagnosed with ADS, aged ≥18 years, and those who were willing to provide informed consent were included in the study, whereas individuals with psychotic disorders, narcotic or drug abusers, were excluded from the study.

### 2.2 Sample Size and Sampling Techniques

The sample size for this study was determined using the formula *n* = *Z* ^2^. *p* *q*/*d* ^2^, where *n* represents the desired sample size, *z* is the standard normal deviation at the required confidence level (1.96), *p* is the proportion of the population (6.5%) [12], *q* is 1‐*p*, and d is the level of accuracy (0.05). Thus, the calculated sample size was obtained to be 96. Convenience sampling technique was used for this study.

### 2.3 Data Collection

Data were collected after obtaining approval from the rehabilitation center and informed consent from the participants. A face‐to‐face interview was conducted using a semi‐structured questionnaire, which included sociodemographic information, alcohol consumption patterns, ADS, and alcohol withdrawal severity. These were assessed using a pre‐validated data collection form, the Alcohol Use Disorder Identification (AUDIT) [8] questionnaire, and the PAWSS [9]. Each participant was interviewed individually in the medication room for approximately 10–15 min, maintaining a friendly and supportive environment. In addition to interview data, clinical symptoms, medication details, and relevant laboratory parameters were obtained from the Kardex (medical records). Data collection was carried out over a period of 3 months to reach the desired sample size. Furthermore, alcohol withdrawal symptoms were reassessed 14 days after the initiation of treatment, and liver enzyme data were collected 30 days after the start of treatment. The questionnaires used in the interviews were initially translated from English to Nepali and then verified through back‐translation to ensure linguistic and conceptual accuracy. The Nepali version of the questionnaire was pilot tested on approximately 10% of the total sample population (*n* ≈ 10), who were not included in the final data analysis. All interviews were conducted in the Nepali language, and responses were recorded on a structured data collection sheet. The internal consistency of the tools was evaluated using Cronbach's alpha, which demonstrated good reliability, with values of 0.87 for the AUDIT and 0.89 for the PAWSS in this study.

### 2.4 Measurements

ADS, alcohol use pattern, alcohol withdrawal severity, and medications prescribed for the management of ADS were considered outcome variables in this study. The ADS was identified on the basis of clinical diagnoses recorded during the rehabilitation period. The AUDIT, a validated 10‐item screening tool developed by the World Health Organization (WHO), was used to assess alcohol consumption, drinking behaviors, and related problems [8]. The tool begins with a 3‐item AUDIT‐C screen, followed by seven additional questions if the AUDIT‐C score is ≥5. Each item is rated on a scale from 0 to 4, producing a total score ranging from 0 to 40, with higher scores indicating a greater risk of alcohol dependence. In this study, AUDIT scores were analyzed both as continuous variables and categorized for clinical interpretation. Similarly, PAWSS [9] was used to evaluate the risk and severity of alcohol withdrawal symptoms among patients undergoing treatment. It consists of three sections: Part A (threshold criteria), Part B (patient interview), and Part C (clinical evidence). A PAWSS score of ≥4 was considered indicative of high risk for complicated alcohol withdrawal syndrome (AWS). PAWSS scores were recorded both before and after treatment to assess the effectiveness of interventions during rehabilitation. Medications prescribed were classified according to the Anatomical Therapeutic Chemical (ATC) classification system, and details such as their generic names, dosages, and frequencies were documented [13].

Sociodemographic variables consisted of age, gender, religion, ethnicity, educational status, and occupation; lifestyle‐related variables included the history of chronic heavy drinking, family history of alcohol use, symptoms of ADS, laboratory parameters, and the duration of stay in the rehabilitation center were the independent variables.

### 2.5 Statistical Analysis

Descriptive statistics were used to summarize the sociodemographic characteristics and alcohol consumption and dependence of the participants, including means and standard deviations (mean ± SD) for continuous variables and frequencies and percentages for categorical variables. The normality of continuous data was assessed using the Shapiro–Wilk test, along with skewness and kurtosis to evaluate the symmetry of the distributions. A paired *t*‐test was conducted to examine changes in alcohol withdrawal severity and liver enzymes at the time of admission and follow‐up. Medication was prescribed based on the severity of ADS, symptoms, and PAWS severity before and after treatment, and they were analyzed using an independent *t*‐test. All statistical analyses were performed in R (Version 4.0.4) and SAS (Version 9.4). For bar graphs and visualizations, the ggplot2 package in R (Version 4.0.4) was used. All significance tests were two‐sided at the 0.05 significance level. As this analysis was exploratory, no adjustments were made for multiple comparisons.

### 2.6 Ethics

Ethical approval for this study was obtained from the Institutional Review Committee of CiST College (Ref. no: 26/080/081), and data collection approval was granted by the Amrita Foundation Rehabilitation Center. Participants were fully informed about the study's purpose and procedures before data collection and were assured of the confidentiality and privacy of their information. Participation was entirely voluntary, and participants had the right to withdraw from the study at any time without any negative consequences. To minimize discomfort, interviews were conducted in a friendly and supportive environment, ensuring no risks were posed to the participants. Confidentiality was maintained by coding the data, and participants’ identities were not disclosed to any third party. Additionally, participants were informed that they could access the study findings upon completion.

## 3 Results

Table 1 provides an overview of the sociodemographic characteristics of ADS patients at the rehabilitation center (*n* = 96). Most of the patients were male (69.0%) and fell within the age range of 31–50 years (69.8%), with a mean age of 37.79 ± 9.61 years. Most of the patients were married (80.2%), and more than one‐third were employed in private jobs (36.5%) or in agriculture (34.4%). Educational background varied, with equal proportions of patients having primary education (24.0%), secondary education (24%), or having no formal education at all (24.0%). Nearly half of the patients belonged to the Janajati community (46.9%), and most identified as Hindu (69.8%). Regarding the duration of the stay at the center, 61.5% was admitted for less than 5 months, 35.4% for 5–10 months, and only 3.1% for 11 months or more.

**TABLE 1:** Sociodemographic characteristics of alcohol dependence syndrome patients at rehabilitation center (*n* = 96).

Characteristics	Category	*n* (%)
**Gender**	Male	66 (69.0)
	Female	30 (31.0)
**Age (years)**	18–30	20 (20.8)
	31–50	67 (69.8)
	≥51	9 (9.4)
	Mean ± SD	37.79 ± 9.611
**Marital status**	Married	77 (80.2)
	Unmarried	12 (12.5)
	Widowed	2 (2.1)
	Separated	2 (2.1)
	Divorced	3 (2.1)
**Occupation**	Government job	7 (7.3)
	Private job	35 (36.5)
	Agriculture	33 (34.4)
	Homemaker	8 (8.3)
	Retired	2 (2.1)
	Unemployed	11 (11.5)
**Education**	Primary	23 (24.0)
	Secondary	23 (24.0)
	Higher secondary	22 (22.9)
	Undergraduate and above	4 (4.2)
	Illiterate	23 (24.0)
**Ethnicity**	Brahmin	37 (38.5)
	Chettri	10 (10.4)
	Dalit	4 (4.1)
	Janajati	45 (46.9)
**Religion**	Hindu	67 (69.8)
	Buddhist	21 (21.9)
	Christian	8 (8.3)
**Duration of rehab admission (month)**	<5	59 (61.5)
	5–10	34 (35.4)
	≥11	3 (3.1)

Abbreviation: SD, standard deviation.

Table 2 depicts that more than one‐third of the patients had been consuming alcohol for 1–5 years (39.6%) or 6–9 years (35.4%), and whiskey emerged as the most commonly consumed alcoholic beverage (84.4%), followed by homemade alcohol (83.3%). Most of the patients reported drinking alcohol four or more times a week (72.9%), with 69.6% consuming 10 or more units per session. The primary reasons for alcohol consumption were family and peer pressure (89.6%) and the pursuit of pleasure (76.0%). The mean AUDIT score was 21.73 ± 2.16. More than half of the patients (58.0%) were classified as having mild alcohol dependency. Notably, the majority (96.0%) were classified as problematic drinkers, whereas only 4.0% were non‐problematic. In accordance with the PAWSS, all patients (100%) were determined to be at high risk for alcohol withdrawal.

**TABLE 2:** Characteristics of alcohol consumption and dependence in rehabilitation center patients (*n* = 96).

Characteristics	Category	*n* (%)
**Duration of alcohol consumption (in years)**	1–5	38 (39.6)
	6–9	34 (35.4)
	≥10	24 (25.0)
**Types of alcoholic beverage dependence** ^a^	Whiskey	81 (84.4)
	Wine	16 (16.7)
	Beer	31 (32.3)
	Homemade alcohol	80 (83.3)
	Homemade malt	37 (38.5)
**Frequency of alcohol consumption**	4 or more times a week	70 (72.9)
	2/3 times a week	23 (24.0)
	2/4 times a month	3 (3.1)
**Quantity of alcohol consumed**	≥10 U	67 (69.6)
	7–9 U	10 (10.4)
	5–6 U	6 (6.3)
	3–4 U	13 (13.5)
**Reason for consuming alcohol** ^a^	Family and peer pressure	86 (89.6)
	Feeling depressed	51 (53.1)
	Stressful life events	30 (31.3)
	Pleasure	73 (76.0)
	Religious belief	29 (30.2)
	Anxiety	9 (9.4)
	Others	5 (5.2)
**Family history to consume alcohol**	None	29 (30.2)
	Father	56 (83.6)
	Mother	11 (16.4))
**Alcohol dependency severity**	Very mild	4 (4.0)
	Mild	56 (58.0)
	Moderate	34 (36.0)
	Severe	2 (2.0)
**AUDIT score**	Mean ± SD	21.73 ± 2.16
**Problematic consumers**	Non‐problematic drinker	4 (4.0)
	Problematic drinker	92 (96.0)
**Alcohol withdrawal severity risk** ^b^	Low risk (0–3)	0 (0.0)
	High risk (≥4)	96 (100.0)

^a^ Multiple response.

^b^Alcohol withdrawal severity risk was measured by Predictive alcohol withdrawal severity scale (PAWSS).

**FIGURE 1:**
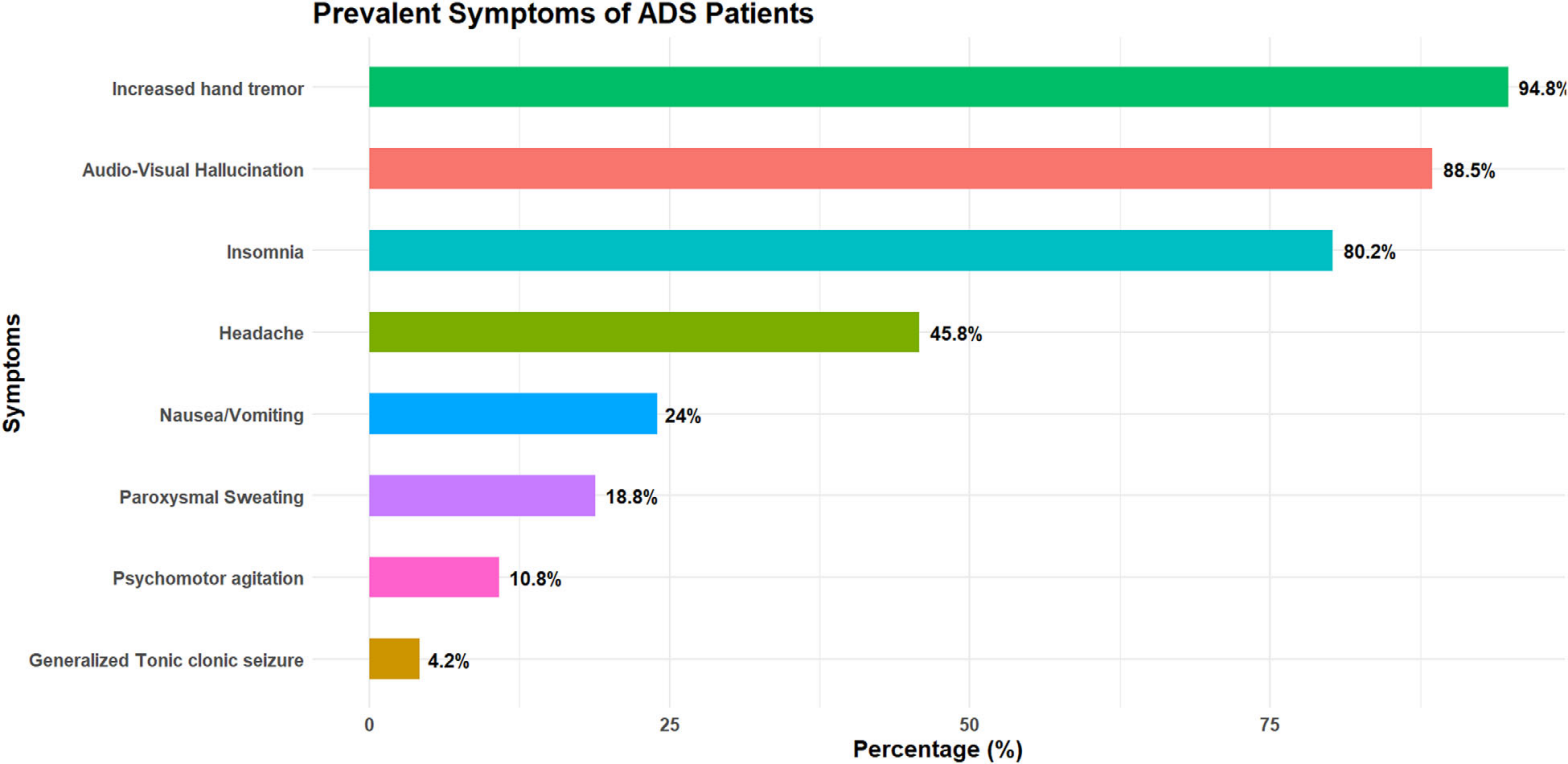
Prevalent symptoms of ADS patients. ADS, alcohol dependence syndrome.

Figure 1 shows the prevalent symptoms observed among patients with ADS. The most frequently reported symptom was increased hand tremors (94.8%), followed by audiovisual hallucinations (88.5%) and insomnia (80.2%), whereas less commonly reported symptoms included psychomotor agitation (10.8%) and generalized tonic–clonic seizures (4.2%).

Table 3 presents the distribution of commonly prescribed medications in ADS. Lorazepam was the most frequently prescribed drug (98.8%), followed by thiamine (97.9%), vitamin B complex (65.6%), and olanzapine (64.6%). Among those prescribed lorazepam, most (74.0%) received a 2‐mg dose, commonly administered three times daily (54.2%). Thiamine was predominantly prescribed at a 100‐mg dose (97.9%), with a typical dosing schedule of twice daily (54.3%). For vitamin B complex, 74.6% were prescribed a 15‐mL syrup, with dosing schedule of thrice daily (61.9%) or twice daily (27.0%). In case of olanzapine, 77.4% of patients received a 5‐mg dose, generally taken once daily (79.0%). Approximately 37% patients were prescribed chlordiazepoxide, with the majority (97.1%) recommended a once daily dosing and 60.0% receiving a 25‐mg dose. Escitalopram was prescribed to 22.5% of patients, most commonly at a 5‐mg dose taken once daily (77.3%). Ursodeoxycholic acid (UDCA) was prescribed to 16.7% of patients, with 81.3% receiving a 300‐mg dose, although 16.7% took it twice daily. Other medications, including benzhexol, fluoxetine, sodium valproate, and others, were prescribed less frequently, typically with a once‐daily dosing schedule.

**TABLE 3:** Medication treatment regimens for patients with alcohol dependence syndrome (ADS).

S. no.	Medication	ATC code	*n* (%)	Dose	*n* (%)	Frequency	*n* (%)
1	Lorazepam	NO5BA56	83 (98.8)	1 mg	13 (15.5)	Once daily	5 (6.0)
				2 mg	71 (74.0)	Twice daily	26 (31.3)
						Thrice daily	52 (54.2)
2	Olanzapine	NO5AH53	62 (64.6)	5 mg	48 (77.4)	Once daily	49 (79.0)
				10 mg	14 (22.6)	Twice daily	13 (21.0)
3	Vit. B complex	A11E	63 (65.6)	Tab	16 (25.4)	Once daily	7 (11.1)
				Syrup (15 mL)	47 (74.6)	Twice daily	17.7 (27.0)
						Thrice daily	40.6 (61.9)
4	Thiamine	A11DA01	94 (97.9)	100 mg	94 (100.0)	Once daily	6 (6.4)
						Twice daily	51 (54.3)
						Thrice daily	37 (39.4)
5	Escitalopram	N06AB10	22 (22.5)	5 mg	20 (90.9)	Once daily	17 (77.3)
				10 mg	2 (9.1)	Twice daily	5 (22.7)
6	Ursodeoxycholic acid	A05AA02	16 (16.7)	150 mg	3 (18.7)	Twice daily	16 (16.7)
				300 mg	13 (81.3)		
7	Benzhexol	N04AA01	7 (7.3)	2 mg	7 (7.3)	Once daily	7 (7.3)
8	Fluoxetine	N06CA03	3 (3.1)	10 mg	3 (3.1)	Once daily	3 (3.1)
9	Sodium Valproate	N03AG01	11 (11.5)	200 mg	7 (7.3)	Once daily	7 (7.3)
—				300 mg	4 (4.2)	Twice daily	4 (4.2)
10	Chlordiazepoxide	N05BA02	35 (36.5)	10 mg	14 (40.0)	Once daily	34 (97.1)
				25 mg	21 (60.0)	Twice daily	1 (2.9)
11	Baclofen	M03BX01	2 (2.1)	10 mg	2 (21.1)	Once daily	2 (2.1)
12	Carbamazepine	N03AF01	2 (2.1)	500 mg	2 (21.1)	Once daily	2 (2.1)
13	Propranolol	C07AA05	4 (4.16)	10 mg	4 (4.16)	Once daily	4 (4.16)
14	Chlorpromazine	N05AA01	2 (2.1)	25 mg	2 (2.1)	Once daily	2 (2.1)
15	Sertraline	N06AB06	1 (1.2)	25 mg	1 (1.2)	Once daily	1 (1.2)
16	Risperidone	N05AX08	3 (3.12)	3 mg	3 (3.12)	Once a day	3 (3.12)
				4 mg	1 (1.2)	Once a day	1 (1.2)
17	Aripriprazole	N05AX12	1 (1.2)	10 mg	1 (1.2)	Once daily	1 (1.2)
18	Diazepam	N05BA01	1 (1.2)	10 mg	1 (1.2)	Once daily	1 (1.2)

Abbreviation: ATC, anatomical therapeutical classification.

Table 4 depicts a significant reduction in the severity of alcohol withdrawal symptoms following treatment. The total severity score decreased from 7.06 ± 0.89 at admission to 3.64 ± 0.94 at follow‐up, indicating a substantial clinical improvement.

**TABLE 4:** Prediction of Alcohol Withdrawal Severity Scale (PAWSS) scores at the time of admission and follow‐up (*n* = 96).

S. no.	Question	At the time of admission (mean ± SD)	Follow up (mean ± SD)
1	Recently intoxicated/drunk (last 30 days)?	0.99 ± 0.102	0.06 ± 0.243
2	Ever undergone alcohol rehabilitation or treatment?	0.93 ± 0.261	0.31 ± 0.466
3	Any previous episodes of alcohol withdrawal?	0.92 ± 0.278	0.20 ± 0.401
4	Ever experienced blackouts?	0.12 ± 0.320	0.05 ± 0.223
5	Ever experienced alcohol withdrawal seizures?	0.91 ± 0.293	0.43 ± 0.497
6	Ever experienced delirium tremens (DTs)?	0.11 ± 0.320	0.11 ± 0.320
7	Combined alcohol with “downers” (e.g., benzodiazepines, barbiturates) in the last 90 days?	0.61 ± 0.489	0.39 ± 0.489
8	Combining alcohol with other substances of abuse in the last 90 days?	0.51 ± 0.503	0.39 ± 0.489
9	Blood alcohol level (BAL) ≥200 on presentation?	0.00 ± 0.00	0.00 ± 0.00
10	Evidence of increased autonomic activity (e.g., HR >120 bpm, tremor, sweating, agitation, nausea)?	0.96 ± 0.201	0.69 ± 0.466
Total score (mean ± SD)		**7.06** ± **0.89**	**3.64** ± **0.94**
Risk level		**Higher risk**	**Moderate risk**

**FIGURE 2:**
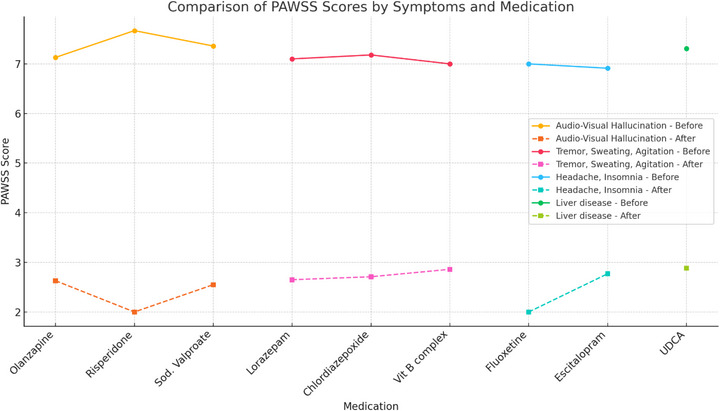
Comparison of medication use with symptoms and PAWS severity before and after treatment. PAWSS, Prediction of Alcohol Withdrawal Severity Scale; UDCA, ursodeoxycholic acid.

Figure 2 illustrates the comparison of PAWSS scores before and after treatment, categorized by medications and associated symptoms. The figure highlights the relative effectiveness of various pharmacological agents in reducing withdrawal severity. Notably, antipsychotic medications such as olanzapine, risperidone, and sodium valproate, primarily prescribed to manage audiovisual hallucinations, showed marked reduction in PAWSS scores posttreatment, with risperidone exhibiting the most significant drop. BZDs, including lorazepam and chlordiazepoxide, are predominantly used to address symptoms such as hand tremors, paroxysmal sweating, and psychomotor agitation, also demonstrated substantial efficacy, indicated in significantly lower posttreatment PAWSS scores. In contrast, antidepressants like fluoxetine and escitalopram, prescribed to alleviate headache and insomnia, produced only mild reductions in severity, which was not statistically significant in this sample. Vitamin B complex, often used as supportive therapy, and UDCA, administered in cases with liver‐related complications also contributed to moderate improvements in PAWSS scores.

Table 5 illustrates significant reduction in liver enzyme levels following treatment (*p* < 0.001).

**TABLE 5:** Distribution of changes in liver enzymes before and after treatment.

Liver enzymes				
	*n* (%)	Before treatment Mean ± SD	After treatment	*p* value
**ALP**	29 (28)	161.69 ± 79.084	89.90 ± 51.109	<0.001
**SGPT**	72 (71.3)	156.60 ± 75.59	55.20 ± 25.944	<0.001
**SGOT**	72 (71.3)	159.50 ± 72.959	62.20 ± 30.77	<0.001
**Bilirubin**	17 (16.8)	1.6940 ± 0.59	1.33 ± 0.445	<0.001

Abbreviation: ALP, alkaline phosphatase.

## 4 Discussion

This study assessed the pharmacological management of ADS among patients at a rehabilitation center in Kathmandu, Nepal. Lorazepam, followed by thiamine, vitamin B complex, and olanzapine, were the most commonly prescribed medications, and their use was associated with a significant reduction in alcohol withdrawal severity and liver enzyme levels.

Alcohol withdrawal represents a critical initial phase in the treatment of ADS, and when alcohol consumption is abruptly stopped, withdrawal symptoms such as tremors, audiovisual hallucinations, insomnia, nausea/vomiting, psychomotor agitation, and seizures can precipitate [14, 15]. In this study, lorazepam 2 mg was the most frequently prescribed medication, commonly administered thrice daily. The widespread use of BZDs, including lorazepam and chlordiazepoxide, is well supported by international guidelines due to their anxiolytic, sedative, and anticonvulsant properties [2, 16, 17, 18]. These agents act by modulating GABA‐mediated central nervous system hyperactivity associated with alcohol withdrawal [15]. Similar prescribing patterns have been reported in neighboring India, where BZDs, particularly lorazepam, are preferred because of its favorable safety profile in patients with hepatic impairment [19].

Thiamine and vitamin B complex were routinely prescribed to prevent Wernicke's encephalopathy and address nutritional deficiencies commonly observed in chronic alcohol users [20]. The predominant use of thiamine at a 100‐mg dose, administered mainly twice daily, is in accordance with WHO and NICE recommendations [2, 21], reflecting adherence to evidence‐based treatment protocols.

The use of atypical antipsychotics such as olanzapine and risperidone aligns with previous evidence supporting their role as adjunctive agents in improving psychiatric comorbidities and reducing cravings during alcohol withdrawal [22]. The significant reduction in PAWSS scores posttreatment, particularly among patients receiving both antipsychotics and BZDs, further supports their clinical utility in this context. Notably, antidepressants such as fluoxetine and escitalopram were prescribed infrequently and primarily for associated symptoms like headache and insomnia, despite their less pronounced effect on withdrawal severity. Although antidepressants are recommended for treating comorbid depression in patients with alcohol use disorder, they have limited efficacy in reducing alcohol consumption or preventing relapse in the absence of depressive symptoms [23].

Medication safety is a critical component of pharmacological management in patients with ADS, as patients with substance use disorders are at greater risk of medication‐related harm due to impaired cognition, hepatic dysfunction, polypharmacy, and possible continued alcohol use. Adequate patient knowledge of prescribed medications, including their purpose, proper dosing, and ADR, is essential to promote safer medication use and adherence. In addition, potential drug–drug and alcohol–medication interactions are a major concern, particularly with central nervous system acting agents, including BZDs, antipsychotics, and antidepressants commonly used in ADS [24, 25, 26]. Concomitant alcohol consumption may exacerbate sedation, cognitive impairment, respiratory depression, and hepatotoxicity. Recent studies highlight that structured medication counseling and patient‐centered education significantly improve medication safety and treatment outcomes in substance use disorder populations [27].

A significant reduction in liver enzyme levels was observed following treatment, indicating sustained abstinence and suggesting a possible hepatoprotective effect of UDCA. Although not universally included in detoxification guidelines, the benefit of UDCA in improving hepatic markers has been supported by meta‐analytic evidence [28]. Furthermore, the marked reduction in PAWSS scores (from 7.06 to 3.64) confirms the effectiveness of early pharmacological interventions in mitigating withdrawal severity, consistent with findings [29].

This study provides valuable insights into the pharmacological management and treatment outcomes of patients with ADS and, to the best of our knowledge, may be the first of its kind conducted in Nepal. Despite its strength, this study has several limitations. The single‐center design and relatively small sample size limit the generalizability of the findings. The use of convenience sampling and reliance on self‐reported data introduce potential risks of selection, recall, and social desirability biases. Additionally, the exclusion of individuals with psychiatric comorbidities and long‐term follow‐up restricts the applicability of findings to broader clinical settings.

## 5 Conclusion

The study highlighted that the use of evidence‐based medications, including BZDs, antipsychotics, and nutritional supplements, was significantly associated with reductions in alcohol withdrawal severity and liver enzyme levels, indicating favorable treatment outcomes. These findings underscore the potential value of structured treatment protocols in rehabilitation settings. Future multicenter studies with larger sample sizes and long‐term follow‐up are recommended to enhance the generalizability of these findings and to support the development of standardized national treatment guidelines for ADS in Nepal.

## Author Contributions

**Sanjay Singh** and **Nirmal Raj Marasine**: conceptualization, project administration, resources. **Sanjay Singh**, **Nirmal Raj Marasine**, and **Sabina Sankhi**: methodology, data curation, validation, formal analysis. **Nirmal Raj Marasine** and **SabinaSankhi**: visualization, writing – original draft, writing. **Gajendra Bahadur Bhuju**: supervision. **Sanjay Singh** and **Nirmal Raj Marasine**, **Sabina Sankhi**, **Kabita Bhandari**, and **Gajendra Bahadur Bhuju**: writing – review and editing.

## Funding

The authors have nothing to report.

## Conflicts of Interest

The authors declare no conflicts of interest.

## Data Availability

The raw data used to support the findings of this study are made available from the corresponding author upon reasonable request.
